# Social Support and Strain Across Close Relationships: A Twin Study

**DOI:** 10.1007/s10519-018-9899-x

**Published:** 2018-04-12

**Authors:** Julia Kutschke, May-Bente Bengtson, Teresa E. Seeman, Jennifer R. Harris

**Affiliations:** 1 Medical Department, Vestfold Hospital Trust, Tønsberg, Norway; 2 Division of Geriatrics, David Geffen School of Medicine, University of California, Los Angeles, CA USA; 3 Division of Health Data and Digitalization, Center for Fertility and Health and Department of Genetics and Bioinformatics, The Norwegian Institute of Public Health, PO Box 4404, 0403 Oslo, Norway

**Keywords:** Twins, Social support, Social strain, Social relationships, Genes and environment, Heritability

## Abstract

Social relationships play a critical role in health and well-being throughout life. We analyzed the genetic and environmental variance co-variance structure for social support and strain across four sets of relationships including with one’s co-twin, spouse/partner, family and friends. The sample included 5288 Norwegian twins aged 40–80. Older people reported less support from their co-twin and friends and less strain from their family and friends. Genetic influences contribute importantly to variation across all the measures, with estimates ranging from 0 to 58%; variance due to shared environmental influences was most important for the twin-relationship, ranging from 0.11 to 0.42%. Social support was negatively correlated with social strain across all sets of relationships. With the exception of the co-twin relationship, these associations were primarily mediated by genetic and non-shared environmental effects.

**Electronic supplementary material:**

The online version of this article (10.1007/s10519-018-9899-x) contains supplementary material, which is available to authorized users.

## Introduction

A large and compelling literature demonstrates the importance of social relationships for physical and mental health throughout life (Cacioppo et al. 2006; Gariépy et al. 2016; House et al. 1988; Ryff and Singer 2005; Uchino et al. 1996). The theoretical underpinnings for this research stem from diverse theories (i.e. theories of social integration, attachment, social networks) spanning several disciplines, including sociology, psychoanalysis, anthropology, and epidemiology. Many of the early studies focused on quantitative aspects of social relationships such as numbers of close connections and membership in various types of groups (i.e. religious, hobby, volunteer) to assess social networks. Although different terminology has been used (social ties, social connectedness) the underlying goal was to provide measures of social embeddedness and social integration (Berkman et al. 2000). Later research, spearheaded by studies in health psychology and social epidemiology, emphasized qualitative (positive and negative valences) aspects of social relationships, including the importance of social support and social strain (House 1981; Sarason et al. 1990).

In light of the powerful effects that social relationships have on physical and mental health, the nature of one’s social relationships is increasingly considered an important health behavior. While many empirical investigations now focus on identifying biological pathways through which social relationships exert their effects on health, virtually no research has examined heritable effects on variation in perceptions of social support and social strain across close relationships. To fill this knowledge gap, we study the genetic and environmental variance–covariance structure across four key relationship realms using the classical twin design. This information is important on two fronts. First, it will help elucidate issues of causality between social support, social strain and specific types of health outcomes where the same sets of genetic or environmental factors may underlie the observed associations. Second, it can help inform the emerging area of behavioral intervention programs that focus on the formation of social relations.

Social support and strain have long term consequences for health and development (Cohen and Wills 1985; Uchino et al. 1996). Social integration and engagement are associated with lower biological risk profiles in adults (Botha et al. 2007; Seeman and McEwen 1996; Seeman et al. 2002b), reduced risks for cognitive and physical decline (Bassuk et al. 1999; Eisenberger and Cole 2012; Fratiglioni et al. 2000; Seeman et al. 2001), lower risks for disease and disability, greater longevity (Holt-Lunstad et al. 2010; Seeman and Crimmins 2001), and protection against major depression (Gariépy et al. 2016; Kendler et al. 2005). Experimental data complement these findings and demonstrate that being in socially supportive relationships buffers against illness and improves resilience to physical and emotional stress (Epel et al. 2004; Kiecolt-Glaser et al. 1997).

While social support has beneficial effects for health, social strain leads to poorer physical health and these effects are stronger than those associated with support (Brooks et al. 2014; Seeman and McEwen 1996; Seeman et al. 2011). Individuals experiencing less social support and with fewer social ties are more likely to have higher average blood pressure and poorer metabolic profiles (Seeman et al. 2010; Uchino et al. 1996), higher levels of cortisol, epinephrine, and norepinephrine (Seeman and McEwen 1996; Seeman et al. 2002a), higher levels of inflammation (as indexed by CRP) (Loucks et al. 2006), greater overall allostatic load (Friedman et al. 2014; Gruenewald et al. 2012), and poorer diurnal cortisol regulation (Friedman et al. 2012) that can slow physiological responses to various stress stimuli. Moreover, research indicates that strain in the partner relationship—which is a central relationship in adulthood—is considered a chronic social stressor that can have a damaging effect on cardiovascular, endocrine and immune functioning (Robles and Kiecolt-Glaser 2003). The negative effects of strain in social relationships are sizeable and confer as great a risk to health and mortality as do the most important known risk factors such as smoking, obesity, high blood pressure and sedentary lifestyle (Holt-Lunstad et al. 2010; House et al. 1988).

Twin studies provide an optimal analytic design for estimating the relative importance of genetic and environmental influences on differences in the way in which individuals experience support and strain in their social relationships. This approach provides results that complements other types of studies. For example, a highly replicated finding in behavioral genetic studies is that many measurements of the environment are influenced, at least in part, by genetic effects. An important implication of this for the study of social relationships, which are often considered measures of the environment, is that individuals play an important role in the shaping of these putative environmental measures. Yet, such studies in the realm of social support and social strain are scarce; the few that exist have focused primarily on the relationship between support/strain with other outcomes. Three of these studies analyzed data from the Mid-life in the US (MIDUS) study (Brim et al. 2004) and only one of those focused explicitly on the variance structure of support and strain in relationships with family and friends (Isaeva et al. 2014). Those results indicated different patterns of influence across the relationship measures, with genetic effects significant only for family support and explaining approximately 38% of the variance. However, these findings should be interpreted with caution due to the small sample and limited power. The other two MIDUS-based twin studies focused on the degree to which common sets of genetic and environmental influences explain the relationship between measures of support and well-being. Schnittker (2008) combined family support, friends support and marital status into a single latent measure of support and then explored the tri-variate relationship between this support measure, happiness and socio-economic position. Results indicated common genetic effects across all three measures but unique sets of environmental influences. Horwitz et al. (2015) found that the relationship between friends support and psychological distress is explained by a common genetic factor that is independent of genetic effects on family support. Finally, a study based on data from the Swedish Adoption Twin Study on Aging (SATSA) assessed social support as the quantity of social relationships and the perceived adequacy of support. Their findings revealed divergent patterns of effects for these measures and showed that genetic influences were important sources of variation only for perceived adequacy of support (Bergeman et al. 1990).

Most research exploring social support and strain focuses primarily on relationships with the family (including spouse/partner) and with friends. However, within the context of twins, family support and strain additionally covers the relationship with the co-twin, which is a unique type of relationship that differs in critical aspects from the relationship with other family members (Segal 1999). More research is needed to elucidate the importance of genetic and environmental influences that affect the nature of social relationships in adulthood. Understanding the twin-relationship is a critical element of this but, to the best of our knowledge, perceptions of support and strain within the twin relationship have not been analyzed in parallel with measures of support and strain from spouse, family and friends. The purpose of this study, therefore, is to analyze the genetic and environmental variance–covariance structure of support and strain across four key relationship domains in a Norwegian twin population and to investigate age and sex effects.

## Methods

### Sample

The data were collected as part of a study on social factors and health (Kutschke et al. 2016) using a mail-out questionnaire sent in 2014–2015. The full study sample included 5442 twins who are participants in the Norwegian Twin Registry (Nilsen et al. 2012). There were 1986 complete pairs and 1470 single responders among the participants. The present study excluded opposite-sex pairs (n = 154 twins) due to the low number of pairs. The resulting sample, 5288 twins, included 1925 complete pairs and 1438 single responders between aged 40–80 years old (mean age = 62.04 years; SD = 9.03). 56.2% of the participants were females. The sample and response rates by sex and zygosity are detailed elsewhere (Kutschke et al. 2016).

### Measures

The questionnaire covered a wide range of areas spanning general health and well-being, specific illnesses, lifestyle factors, socio-economic status, psychosocial factors and several measures of the nature of their relationships with family, friends, and colleagues. The work presented here is based on analyses of the measures that describe social support and strain within four categories of relationships including the co-twin, spouse/partner, family (excluding co-twin and spouse) and friends. These support and strain measures (except the co-twin) were originally described by Schuster et al. (1990) and Walen and Lachman (2000) and revised by the MIDUS study. Scales construction and handling of missing data was conducted as in MIDUS with the exception that MIDUS did not have a separate category of relationship for the co-twin but rather included the co-twin in the questions that asked about the family. In contrast, our questionnaire treated the co-twin and the rest of the family as two separate categories. Thus, the social support measures were constructed independently for each of the following four types of relationships: the co-twin, the spouse/partner, the family (excluding co-twin and spouse) and friends. Each relationship measure was based on four items (as an example we present questions regarding the co-twin): (1) how much does your co-twin really care about you?; (2) how much does he/she really understand the way you feel about things?; (3) how much can you rely on him/her if you have a serious problem?; (4) how much can you open up to him/her if you need to talk about your worries? Each item was scored on a scale ranging from 1 to 4 (1 = a lot, 2 = some, 3 = a little, 4 = not at all) and reverse-coded before calculating the mean score which then was assigned to be the value for co-twin (spouse, family, friends) support. The response rates for these scales were rather high with 96% of the participants having a valid value for all four items of the scales regarding twin, family and friends and 78.3% regarding spouse/partner. However, for a scale score to be computed, it was sufficient that at least one item of the scale was answered. Missing data, as in the MIDUS study, were substituted by the respondent’s mean value. As a result, the calculated social support measures range from 1 to 4 with higher values indicating more support and lower values indicating less support.

In parallel, the scales measuring social strain were based on the following four items (as an example we present the questions regarding the co-twin): (1) how often does your co-twin make too many demands on you?; (2) how often does he/she criticize you?; (3) how often does he/she let you down when you are counting on him/her?; (4) how often does he/she get on your nerves? As the support items, the strain items were also scored from 1 to 4 (1 = often, 2 = sometimes, 3 = rarely, 4 = never), and were reverse-coded before calculating the mean score so that a higher value reflects a higher level of strain. The response rates for the strain scales were similar to those of the support scales (> 96% of the twins had valid values for all four items for the twin, family and friends scales and 77.6% for the spouse/partner scale), and the method of handling missing data in the computation of the social strain values was the same as that used for social support—missing items were replaced by respondent’s mean and their average was assigned as the social strain measure. Again, the scale was calculated only for the cases where a response for at least one item was available.

### Analysis

Descriptive analyses were conducted for the support and strain measures across the four types of relationships for males and females. Intraclass and cross-twin cross-trait correlations were calculated by zygosity and sex to provide insight into the genetic and environmental variance structure of the traits and their covariance.

Biometrical modeling was used to decompose the phenotypic variance into components accounted for by the effects of additive genetic (A), dominant genetic (D), common environmental (C) and unique environmental (E) influences (Neale 2009; Posthuma et al. 2003) and to estimate how these influences contribute to the covariation between support and strain. The estimates were derived from bivariate models specifying a correlated factor structure between support and strain within each of the four types of relationships (co-twin, spouse/partner, family, friends). We estimated the effects of sex and age, the genetic and environmental variance components for each measure plus the sources of covariation between support and strain for each type of relationship. These included the additive genetic (rG), dominant genetic (rD), shared environmental (rC) and specific environmental (rE) correlations.

Prior to constructing the bivariate models between support and strain, we determined which variance components to specify for each measure of support and strain across the four types of relationships. First, we inspected the intraclass and cross-twin cross-trait correlations to determine the most plausible set of effects (ACE or ADE) influencing variation for each measure. Next, to ensure model identification, further constraints were specified so that the genetic and environmental covariation between support and strain could be estimated. This is necessary because rG cannot be estimated simultaneously with rD or rC unless one of these parameters is constrained to a fixed value. Therefore, when ACE was indicated for both support and strain, a series of bivariate models was conducted, separately for each sex, where rG was freely estimated, but rC was fixed to successive values ranging from 0 to − 1.0 with 0.1 step increments. The model with the lowest Akaike Information Criterion (AIC) value was then selected for each sex and the respective values for rC were employed in the full bivariate model. This procedure was not needed for estimating rD because D did not influence variation in both support and strain across any of the four types of relationships (twin, spouse, family, friends).

If the set of effects influencing variation in support differed from those affecting variation in strain (i.e., for example, ACE for support and ADE for strain) then the bivariate model specified ADCE but fixed C or D to zero for the relevant measure and constrained rC and rD to zero.

Finally, the lower boundary of the diagonal elements in the matrices containing the path estimates was set to zero to eliminate symmetrical solutions (with flipped signs of the path estimates) that commonly occur. All models were parameterized for four zygosity groups—MZ males (MZM), DZ males (DZM), MZ females (MZF) and DZ females (DZF)—and allowed means and variances to differ by sex and means to vary with age.

Hypothesis testing was performed by comparing the fits between sub-models that successively drop specific variance components to determine the model which best describes the variance–covariance structure within each type of relationship. The log-likelihood ratio test and AIC were employed to test whether the fit of simplified (restricted) models is significantly worse than of the full model.

The modeling analyses were conducted using the open source statistical software R (R Core Team 2015) in OpenMx package (Boker et al. 2016; Neale et al. 2015; Pritikin et al. 2015) suitable for structural equation modelling.

## Results

Table 1 presents summary statistics of the support and strain measures by sex. The number of missing observations is quite low for most of the scales (< 2% of the total sample size) with the exception of spouse support and strain. This is explained primarily by the high percentage (approximately 20%) of respondents who did not have a spouse/partner at the time of completing the questionnaire. The average score for each scale differed significantly between males and females (p < 0.01) with the exception of spouse and friends strain. The direction of these differences was fairly consistent with women reporting higher levels of support and strain with but men reported higher levels of support from their spouses/partners.

**Table 1:** Descriptive statistics of the relationship measures by sex (total sample size is 5288 twins, 2314 males and 2974 females)

Measure	Sex	n missing (%)	Mean (SD)	Median	Correlation with age (95% CI)	
Twin support^#^						
	M	43 (0.79)	3.33 (0.70)	3.50	− 0.10	(− 0.14; − 0.06)
	F	54 (0.99)	3.60 (0.63)	4.00	− 0.10	(− 0.14; − 0.06)
Spouse support^#^						
	M	306 (5.62)	3.76 (0.42)	4.00	− 0.01	(− 0.05; 0.03)
	F	784 (14.41)	3.66 (0.51)	3.75	− 0.02	(− 0.06; 0.02)
Family support^#^						
	M	30 (0.55)	3.48 (0.54)	3.50	0.03	(− 0.01; 0.07)
	F	58 (1.07)	3.54 (0.52)	3.75	0.04	(0.01; 0.08)
Friends support^#^						
	M	53 (0.97)	3.27 (0.54)	3.25	− 0.09	(− 0.13; − 0.05)
	F	96 (1.76)	3.50 (0.50)	3.50	− 0.08	(− 0.12; − 0.05)
Twin strain^#^						
	M	47 (0.86)	1.56 (0.56)	1.50	− 0.03	(− 0.07; 0.01)
	F	74 (1.36)	1.69 (0.65)	1.50	− 0.05	(− 0.09; − 0.01)
Spouse strain						
	M	311 (5.71)	2.04 (0.57)	2.00	− 0.04	(− 0.08; 0.00)
	F	785 (14.42)	2.03 (0.62)	2.00	− 0.00	(− 0.05; 0.04)
Family strain^#^						
	M	37 (0.68)	1.65 (0.51)	1.50	− 0.08	(− 0.12; − 0.04)
	F	70 (1.29)	1.77 (0.58)	1.75	− 0.11	(− 0.14; − 0.07)
Friends strain						
	M	52 (0.96)	1.56 (0.47)	1.50	− 0.07	(− 0.11; − 0.03)
	F	100 (1.84)	1.56 (0.49)	1.50	− 0.07	(− 0.10; − 0.03)

^#^p < 0.01 for the mean difference by sex

**Fig. 1:**
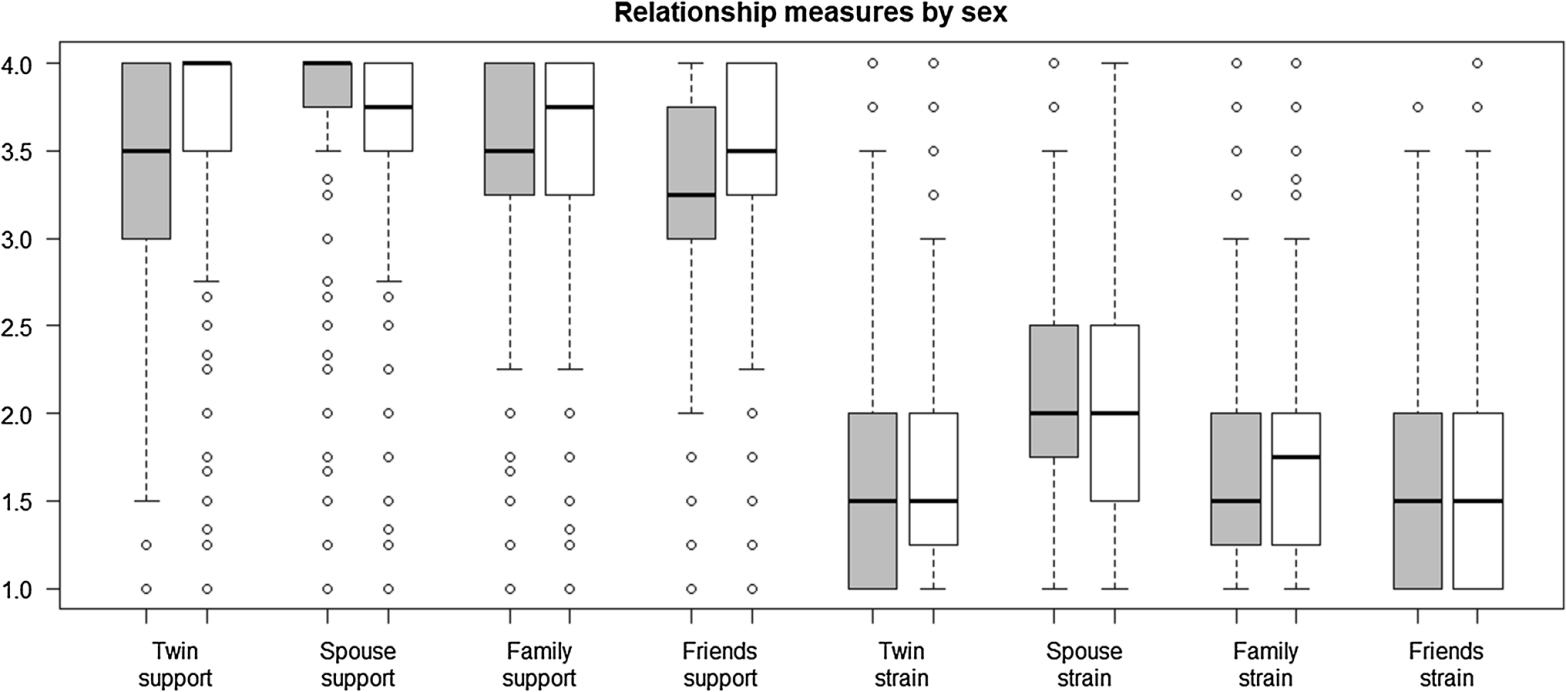
Box plot showing the score distribution of the relationship measures by sex. The horizontal line within each box indicates the median, grey boxes are for males and white boxes are for females

Inspection of the medians of the relationship scales (Table 1) reveals that the distributions are skewed, with many participants reporting high levels of social support and low levels of strain (depicted by box plot in Fig. 1).

Twin support, family strain, and friends support and strain correlated negatively with age (last column in Table 1) indicating that older people reported less support from their co-twin and friends and less strain from their family and friends.

Table 2 contains age-adjusted intraclass correlations by sex and zygosity. MZ values were greater than DZ values across all the measures with the exception of family strain in males, though there is some overlap between the confidence intervals for the MZ and DZ data. The pattern of correlations is generally consistent with the presence of some genetic effects for these measures. For friends support and spouse strain, the MZ correlations were greater than twice DZ correlations indicating that some variation may be explained by dominant genetic effects. Evidence for shared environmental effects was also indicated, particularly for support and strain in the twin relationship, where twice the value of the DZ correlations exceeded the MZ correlations.

**Table 2:** Intraclass correlations (adjusted for age) and 95% confidence intervals for the relationship measures by zygosity and sex

	n pairs	Support				Strain			
		Twin	Spouse	Family	Friends	Twin	Spouse	Family	Friends
MZM	402	0.59	0.27	0.26	0.32	0.53	0.37	0.19	0.22
		(0.53; 0.66)	(0.17; 0.37)	(0.17; 0.35)	(0.23; 0.41)	(0.45; 0.59)	(0.27; 0.46)	(0.09; 0.28)	(0.12; 0.31)
DZM	375	0.41	0.14	0.17	0.10	0.38	0.17	0.20	0.16
		(0.32; 0.49)	(0.02; 0.25)	(0.07; 0.27)	(− 0.00; 0.20)	(0.29; 0.47)	(0.06; 0.28)	(0.10; 0.30)	(0.06; 0.26)
MZF	600	0.69	0.15	0.34	0.39	0.64	0.27	0.39	0.36
		(0.64; 0.73)	(0.04; 0.25)	(0.26; 0.41)	(0.32; 0.46)	(0.59; 0.69)	(0.17; 0.37)	(0.32; 0.46)	(0.29; 0.43)
DZF	547	0.61	0.05	0.20	0.09	0.44	0.09	0.19	0.15
		(0.55; 0.66)	(− 0.06; 0.16)	(0.12; 0.28)	(0.00; 0.18)	(0.36; 0.50)	(− 0.02; 0.20)	(0.10; 0.27)	(0.07; 0.24)

Phenotypic correlations between the measures of support and strain show an inverse relationship; those who score high on a support measure, score low on the respective strain measure. The magnitudes of these correlations are moderate, and significantly weaker for men than for women: − 0.22 (− 0.26; − 0.18) for males and − 0.43 (− 0.46; − 0.39) for females for the twin relationship; − 0.26 (− 0.30; − 0.22) for males and − 0.35 (− 0.39; − 0.31) for females for the family relationship; − 0.17 (− 0.21; − 0.13) for males and − 0.27 (− 0.31; − 0.23) for females for the friends relationship. In contrast, the correlation between spouse support and spouse strain does not differ across sex, the values were − 0.46 (− 0.50; − 0.43) for males and − 0.50 (− 0.53; − 0.46) for females. The general pattern of cross-twin cross-trait correlations (Table 3) also shows greater MZ than DZ values, suggesting that genetic effects may contribute to the covariation between these measures of support and strain. However, as in Table 2, the overlap in confidence intervals for the MZ and DZ estimates makes it difficult to draw strong conclusions about the importance of effects.

**Table 3:** Cross-twin cross-trait correlations by sex and zygosity

	Twin	Spouse	Family	Friends
MZM	− 0.20	− 0.24	− 0.07	− 0.02
	(− 0.30; − 0.08)	(− 0.35; − 0.13)	(− 0.18; 0.04)	(− 0.14; 0.09)
DZM	− 0.12	− 0.12	− 0.11	− 0.06
	(− 0.23; 0.00)	(− 0.23; 0.01)	(− 0.23; 0.01)	(− 0.18; 0.06)
MZF	− 0.44	− 0.17	− 0.16	− 0.10
	(− 0.52; − 0.35)	(− 0.28; − 0.07)	(− 0.26; − 0.05)	(− 0.21; 0.00)
DZF	− 0.27	− 0.09	− 0.07	− 0.02
	(− 0.37; − 0.16)	(− 0.20; 0.02)	(− 0.18; 0.04)	(− 0.13; 0.09)

Based on the intraclass correlations, twin and family relationships were parametrized by an ACE model. After fitting models separately for males and females, the values for the full bivariate model for the twin relationship specified rC = − 0.6 for males and − 0.8 for females. The respective values for the family relationship fixed rC = − 0.8 for males and − 1.0 for females. For the spouse and friends relationships, an ADCE model was specified (ACE for support and ADE for strain for spouse and ADE for support and ACE for strain for friends). In these models rD and rC are fixed to zero.

**Fig. 2:**
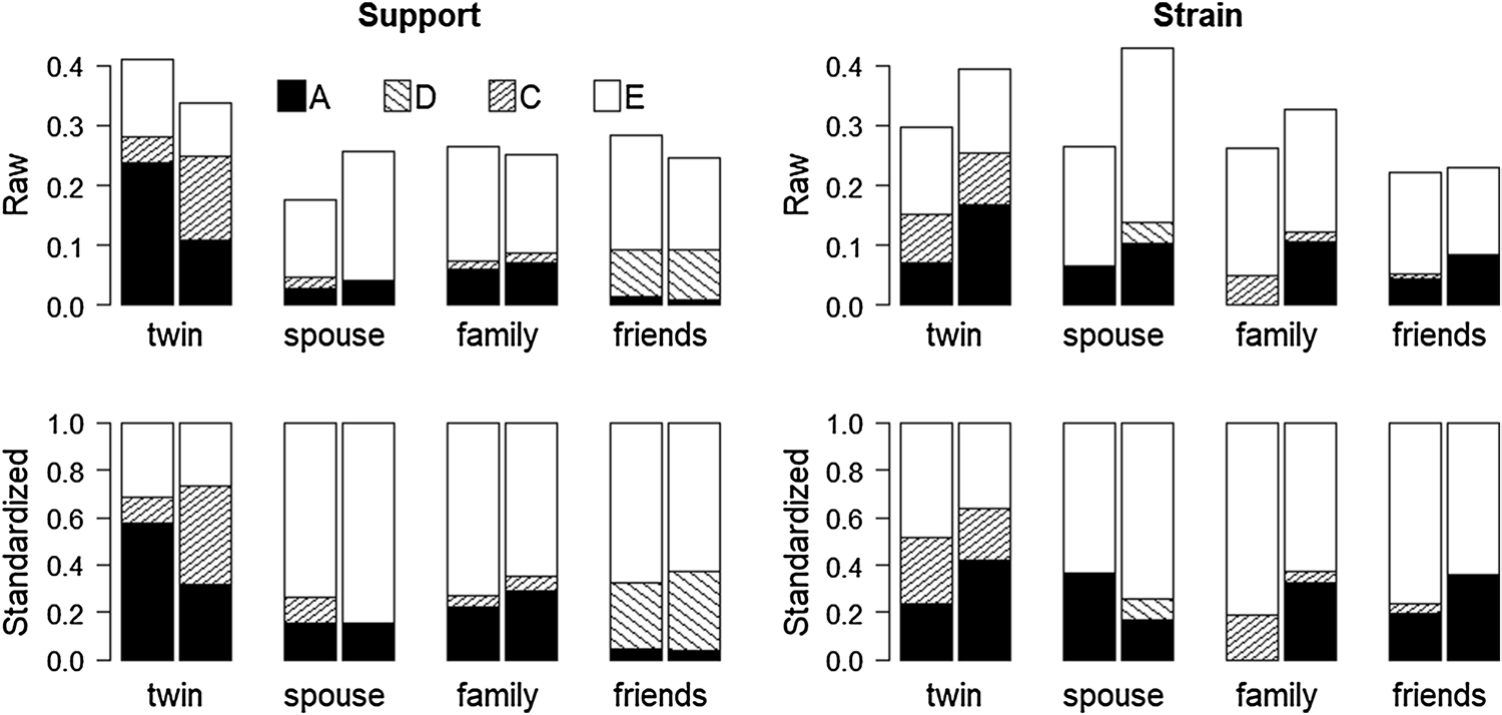
Raw and standardized variance components for the relationship measures by sex. Within each type of relationship, the left bar is for males and the right bar is for females. The values for the variance estimates are based on the full models presented in Table 4 which also provides the 95% confidence intervals

Figure 2 graphs the raw and standardized variance components by sex derived from the full models listed in Table 4. Inspection of the magnitude of the variation across the measures of support for males and females (raw variances in Fig. 2) reveals greater variation among the women only for marital support, and greater variation among the men for the measures of co-twin, family and friends support. In contrast, variation across the measures of strain is greater among women. Raw genetic variance is generally greater for women with the exception of twin support whereas standardized genetic variance (heritability) is larger for men for twin support and spouse strain. For spouse support and friends strain heritabilities appear to be very similar.

**Table 4:** Parameter estimates and (95% confidence intervals) for bivariate models accounting for age effects on means and heterogeneity across sex

	Support						Strain						rG	rD	rC	rE	rP	% of rP explained by			
	Mean	Age	St A	St D	St C	St E	Mean	Age	St A	St D	St C	St E						a	d	c	e
Twin																					
m	3.41 (3.37; 3.45)	− 0.06 (− 0.10; − 0.02)	0.58 (0.41; 0.72)		0.11 (0.00; 0.26)	0.31 (0.27; 0.37)	1.55 (1.52; 1.59)	− 0.02 (− 0.05; 0.02)	0.24 (0.03; 0.45)		0.27 (0.09; 0.44)	0.49 (0.42; 0.56)	− 0.05 (− 0.37; 0.39)		− 0.6	− 0.28 (− 0.37; − 0.19)	− 0.23 (− 0.28; − 0.18)	8		45	48
f	3.65 (3.62; 3.68)	− 0.04 (− 0.07; − 0.01)	0.32 (0.21; 0.43)		0.42 (0.31; 0.51)	0.26 (0.23; 0.30)	1.66 (1.63; 1.70)	− 0.04 (− 0.07; − 0.01)	0.42 (0.29; 0.55)		0.22 (0.10; 0.34)	0.36 (0.32; 0.40)	− 0.37 (− 0.53; − 0.18)		− 0.8	− 0.24 (− 0.31; − 0.17)	− 0.45 (− 0.49; − 0.41)	30		54	16
Spouse																					
m	3.76 (3.74; 3.79)	− 0.00 (− 0.03; 0.02)	0.15 (0.04; 0.15)	0	0.11 (0.00; 0.21)	0.74 (0.64; 0.84)	2.05 (2.02; 2.09)	− 0.02 (− 0.05; 0.02)	0.36 (0.13; 0.36)	0.00 (0.00; 0.00)	0	0.64 (0.55; 0.73)	− 0.80 (− 1.00; − 0.45)	0	0	− 0.42 (− 0.50; − 0.34)	− 0.48 (− 0.52; − 0.43)	39	0	0	61
f	3.65 (3.63; 3.68)	− 0.01 (− 0.03; 0.02)	0.16 (0.03; 0.25)	0	0.00 (0.00; 0.09)	0.84 (0.75; 0.95)	2.04 (2.01; 2.07)	− 0.00 (− 0.03; 0.03)	0.17 (0.06; 0.33)	0.09 (0.00; 0.19)	0	0.74 (0.66; 0.84)	− 0.95 (− 1.00; − 0.54)	0	0	− 0.46 (− 0.53; − 0.39)	− 0.52 (− 0.55; − 0.48)	29	0	0	70
Family																					
m	3.49 (3.46; 3.52)	0.01 (− 0.02; 0.04)	0.23 (0.02; 0.35)		0.05 (0.00; 0.13)	0.73 (0.64; 0.81)	1.66 (1.63; 1.69)	− 0.04 (− 0.07; − 0.01)	0.00 (0.00; 0.00)		0.19 (0.01; 0.26)	0.81 (0.72; 0.88)	− 1.00 (− 1.00; 1.00)		− 0.8	− 0.30 (− 0.37; − 0.21)	− 0.31 (− 0.35; − 0.26)	0		25	74
f	3.55 (3.53; 3.58)	0.02 (− 0.00; 0.04)	0.29 (0.10; 0.42)		0.06 (0.00; 0.21)	0.65 (0.58; 0.73)	1.76 (1.73; 1.78)	− 0.07 (− 0.09; − 0.04)	0.32 (0.15; 0.44)		0.05 (0.00; 0.19)	0.62 (0.56; 0.69)	− 0.45 (− 0.68; 0.03)		− 1	− 0.31 (− 0.38; − 0.24)	− 0.39 (− 0.43; − 0.36)	35		15	51
Friends																					
m	3.28 (3.25; 3.31)	− 0.06 (− 0.09; − 0.03)	0.05 (0.00; 0.37)	0.29 (0.00; 0.37)	0	0.68 (0.59; 0.76)	1.56 (1.53; 1.59)	− 0.03 (− 0.06; − 0.01)	0.19 (0.11; 0.32)	0	0.04 (0.00; 0.21)	0.77 (0.77; 0.86)	− 1.00 (− 1.00; − 0.17)	0	0	− 0.11 (− 0.20; − 0.02)	− 0.17 (− 0.22; − 0.12)	53	0	0	46
f	3.50 (3.47; 3.52)	− 0.05 (− 0.07; − 0.03)	0.04 (0.01; 0.37)	0.33 (0.00; 0.40)	0	0.63 (0.57; 0.70)	1.56 (1.53; 1.59)	− 0.03 (− 0.05; − 0.01)	0.36 (0.21; 0.42)	0	0.00 (0.00; 0.12)	0.64 (0.58; 0.71)	− 0.91 (− 1.00; − 0.24)	0	0	− 0.26 (− 0.33; − 0.19)	− 0.27 (− 0.31; − 0.23)	40	0	0	60

*m* males, *f* females, *St A* standardized additive genetic variance, *St D* standardized dominant genetic variance; *St C* standardized shared environmental variance, *St E* standardized specific environmental variance, *rG* additive genetic correlation, *rD* dominant genetic correlation, *rC* shared environmental correlation, *rE* specific environmental correlation, *rP* phenotypic correlation

Table 4 presents estimates from the bivariate models by sex. As seen by the tabled values, the confidence limits overlap substantially for the estimates of familial effects in several of the models. This is illustrated, for example, for the confidence intervals around estimates of A and C among the males for twin strain, spouse support and family support. Likewise, there is a large overlap between the confidence intervals for A and D for spouse strain in the females and for friends support in both sexes. Lack of precision in the variance estimates greatly affects the power to drop C or D and test for the significance of these parameters in submodels. Therefore, the models specified in Table 4 were retained and used to derive estimates of the covariation between support and strain. As seen from the values in Table 4, the estimates of heritable effects (A or D) varied considerably, ranging from 0% for family strain for men to 58% for twin support for men. Shared environmental influences were greatest for the twin-relationship (ranging from 11% for twin support in males to 42% for twin support in females) and for family strain for males (19%). Variance explained by specific environment is sizeable for all measures (ranging from 26% for twin support to 84% for spouse support).

The estimated genetic and environmental correlations between support and strain for each of the four relationship types (Table 4) were all negative as were the phenotypic correlations between support and strain across each type of relationship. As described in the “Methods” section, rD and rC were fixed at values that provided the best model fit in order to meet model identification requirements. The estimates for rG vary from − 0.05 for the twin relationship in men to − 1.0 for the family and friends relationships in men, however, with the exception of the twin relationship in females [rG = − 0.37 (− 0.53; − 0.18)], these should be interpreted with caution due to wide confidence intervals. rE ranged from − 0.11 for friends in males to − 0.46 for spouse in females and was similar for men and women across the relationship types with the exception of friends where rE was greater for women. The proportion of the phenotypic correlations explained by the genetic and environmental sources of covariance are also presented in Table 4.

Traditional hypothesis testing was also performed by comparing the fits between sub-models that successively drop specific variance components to determine the model which best describes the variance–covariance structure within each type of relationship. Table 5 presents the results of the biometrical modeling describing the starting model, best-fitting model, and respective fit statistics. For the twin relationship, the full model fit the data best. For the spouse, family (females) and friends (males) relationships, dropping C for support resulted in improvement of model fit, and the same was observed for strain for spouse and friends relationship and family in females. In addition, dropping A for strain in males lead to a better model fit for the family relationship. Confidence intervals in the simplified models narrowed for most of the variables (with the exception of spouse and friends support in women and family support in men). Likewise, the confidence limits of the genetic correlations between support and strain improved for all types of relationships among the men. Detailed output of the best-fitting models is presented in Table S1 in the Supplementary material.

**Table 5:** Model fitting results (fit of the full bivariate vs. best-fit model)

	Starting model					Best model				
	Support	Strain	− 2 LL	df	AIC	Support	Strain	Δχ^2^	Δdf	ΔAIC
Twin^a^	ACE_m_ + ACE_f_	ACE_m_ + ACE_f_	11618.06	7533	− 3447.94	ACE_m_ + ACE_f_	ACE_m_ + ACE_f_	–	–	–
Spouse	ACE_m_ + ACE_f_	ADE_m_ + ADE_f_	8485.63	6050	− 3614.37	AE_m_ + AE_f_	AE_m_ + AE_f_	1.45	4	− 6.55
Family^b^	ACE_m_ + ACE_f_	ACE_m_ + ACE_f_	10973.12	7542	− 4110.88	AE_m_ + AE_f_	ACE_m_ + AE_f_	–	–	− 4.12
Friends	ADE_m_ + ADE_f_	ACE_m_ + ACE_f_	10186.10	7450	− 4713.90	AE_m_ + ADE_f_	AE_m_ + AE_f_	1.81	3	− 4.19

All models accounted for age effect on means and heterogeneity across sex

*m* males, *f* females, *− 2LL* − 2 × Log-likelihood, *df* degrees of freedom, *AIC* Akaike’s Information Criterion

^a^The full model for the twin relationship had the best fit

^b^In the full model for the family relationship rC was fixed to − 0.8 for males and to − 1.0 for females. In the best-fitting model, C was fixed to zero for support for both sexes and for strain for females, consequently, rC was also constrained to zero for both sexes. Therefore, the best-fitting model is not nested within the full one

## Discussion

We explored the genetic and environmental variance–covariance structure for measures of social support and social strain across four types of relationships (co-twin, spouse/partner, family and friends) using data from 5288 Norwegian twins. Our main findings revealed that: (1) older people and men tended to report less support and less strain; (2) familial effects and non-shared environment account for a sizable amount of variation in perceptions of support and strain across the relationships; (3) The relative importance of the familial factors (genetic and shared environmental influences) varies considerably and is greatest for the twin relationship; (4) the association between support and strain is primarily mediated by genetic and non-shared environmental influences for the relationships between spouse and friends, whereas shared environmental influences are also important in mediating mediate the relationships between support and strain for the co-twin and family; and (5) non-shared environmental effects are, generally, moderately correlated across support and strain, indicating that there is overlap but also important differences in the idiosyncratic experiences that affect perceptions of support and strain.

### Age and sex effects on the levels of reported support and strain

Older men and women, tended to report less support from their co-twin and friends and less strain for family and friends, in addition, older women reported less strain in their relationship with their co-twin (Table 1). The correlations with age were modest, but significant. In contrast, there were no age differences for support or strain in relationships with the spouse/partner and family or with twin strain among males.

Our pattern of age findings for social strain parallel those from MIDUS (Walen and Lachman 2000) and studies using other indices to measure social networks (English and Carstensen 2014). This pattern is also highly consistent with theories of aging that predict improvements in social relationships with age and the minimization of negative social experiences (Luong et al. 2011). Our age patterns for social support coincide partly with those from MIDUS: analyses of than 2000 participants revealed that family support was positively associated with age and the broader range of support measures was positively associated with age in the subset of individuals (N = 949) aged 34–84 who participated in the biomarker MIDUS 2 study (Brooks et al. 2014). Age was also positively associated with family support both in the subset of MIDUS twins (N = 783 pairs, 1566 individuals) aged 25–74 (Isaeva et al. 2014) and in our data. However, in our sample this correlation was weak and the age effect was not significant in the model. The discrepancy in the age findings between these studies could reflect, in part, age differences in the samples: the Norwegian twins are older (mean age = 62.04 years) than the MIDUS twin sample (mean age = 45.0 years). To explore this further, we also tested for age effects based on groups stratified into 10-year age intervals; however, there was no evidence for age differences between these subgroups (data not shown). We can only speculate about the reasons why age trends in perceptions of social support may differ between the Norwegian and MIDUS samples. Our findings could reflect cultural and contextual factors that differ between the two populations regarding family constellations, healthy aging, as well as expectations and norms for receiving support and reporting strain. For example, Norway is considered to one of the best places in the world to live for seniors, it provides high financial security and is ranked at the top by the Global Age Watch (http://www.helpage.org/global-agewatch/)—a coalition of organizations dedicated to improving living standards for the world’s rapidly growing population older than 60. Thus, expectations for receiving support from close social ties versus state-financed services could be vastly different between the countries.

Distinct lines of research provide complementary perspectives about age-related contexts that would impact experiences of social support in both positive and negative directions. Socioemotional selectivity theory (Carstensen 2006) posits that people increasingly prioritize emotional goals with age and will proactively structure their social networks to maintain their closest social ties and more meaningful interactions. Accordingly, the quantity of social relationships may shrink with age as a result of network pruning, but the most rewarding relationships are retained (Charles and Carstensen 2010; Wrzus et al. 2013). This is supported by longitudinal findings showing that individuals maintain their close social ties with age and, in comparison with younger participants, also report more positive and fewer negative emotions (English and Carstensen 2014).

However, the process of age-related selectivity in the choices made about social relationships is counter balanced by other age-related factors—typically out of one’s control, that disrupt our ability to selectively shape and engage in our social worlds. Examples of such factors include relocation of friends or family to care facilities, illness or death of close friends, loss or caretaking of an ill spouse/partner, and chronic health problems that lead to reduced mobility, hearing loss or other health infirmities. These difficulties can strain relationships and support systems, particularly when they are severe and persistent (Gurung et al. 2003; Krause and Shaw 2002). Furthermore, effects can operate in a reciprocal manner whereby caregiving kindles tensions and strain in both the care provider and the recipient. Evidence shows that family relationships can become less positive and more strained when faced with disability and dependency of an aging parent (Kim et al. 2017), but there are also negative reactions to being helped (Martire et al. 2011; Newsom and Schulz 1998).

Our age-related findings, suggesting less support from the co-twin and friends, but also less strain from family and friends, are consistent with perspectives outlined above, but too preliminary to warrant strong conclusions about the specific effects at play. For example, the age-related decreases in reports of support from friends and the co-twin could reflect age-related increases in disabilities and health conditions that impact individuals—or those with whom they have close relationships, in ways that impinge upon their ability for social engagement (Gurung et al. 2003). Likewise, our results showing age-related decreases in reports of strain with family and friends are congruent with socioemotional selectivity whereby individuals preferentially engage in those relationships that provide the greatest opportunity of fulfilling emotional goals. This would lead to reductions in social strain. But the results are also in agreement with other evidence that older people tend to report less conflict in social relationships and also perceive tension and conflict as less stressful than do younger adults (Luong et al. 2011). We can only speculate about the specific causes of our age-related findings and more conclusive interpretations would require a more comprehensive analysis of the social contexts and health circumstances of the participants.

Compared to men, women reported higher levels of support for all relationships except marital support and higher levels of strain from their twin and their family. There were no sex differences for reports of spouse and friends strain, and the same trends were found when analyzing the MIDUS sample (Matzek and Cooney 2009; Walen and Lachman 2000). Overall, the patterns of age and sex variation in the Norwegian data echo those in national surveys like MIDUS. This suggests that this twin sample provides a good basis to investigate the genetic and environmental sources of variation in perceptions of the quality of their social relationships.

### Variance structure of social support and strain across types of relationships

The variance component analyses revealed fairly similar patterns of effects for the measures of social support and social strain across the different types of relationship. Genetic factors were important across all the measures of support and strain (except family strain in males), accounting for 15 to 58% of the variation. In comparison to the results based on the MIDUS data, the heritability estimates are fairly similar for family support and strain, but larger than MIDUS estimates for the measures of friends support and strain. However, these comparisons are limited because the studies based on the MIDUS twins had little power and sex differences were not estimated. Further, the heritability estimates from two of the MIDUS studies were derived from the twin correlations rather than through biometrical modeling (Horwitz et al. 2015; Schnittker 2008).

Shared environmental influences also contribute importantly to variation in twin support and strain and in family strain for men, with effects ranging between 11 and 42%. Non-shared environmental influences explained a substantial amount of variation (ranging from 26 to 84%) in the measures of support and strain. This is not surprising given that an individual experience of social support and social strain will be contextualized by the cumulative effect and unique constellation of many factors specific to their own life circumstances, health status and the qualitative and quantitative characteristics of their social worlds. Family size, family closeness and family constellations differ between people, as do networks of friends, and the quality of relationships with close others. Thus, the experiential backdrop against which one develops perceptions of social support and social strain in their close relationships is highly idiosyncratic.

Our findings are among the first to highlight the importance of genetic factors for the patterning of social relationships in adulthood. These results have implications for research going forward. The burgeoning evidence showing that social relationships affect health has focused attention on elucidating the mechanisms and pathways through which social relationship factors might act. Important inroads have already been made in identifying hormonal, neural, neuroendocrine, genetic, and immunological profiles associated with social ties and social isolation (Cacioppo et al. 2015; Hawkley and Cacioppo 2010). For example, greater social integration and higher levels of reported emotional support are associated with lower levels of major stress hormones (e.g. cortisol, norepinephrine, and epinephrine) (Friedman et al. 2012, Seeman et al. 1994; Seeman and McEwen 1996), lower levels of the pro-inflammatory cytokine interleukin-6 (IL-6) and C-reactive protein (Glei et al. 2012), lower overall levels of allostatic load (Brooks et al. 2014; Seeman et al. 2002b, 2014) and better cardiovascular, immune and hormone profiles (Heaphy and Dutton 2008). Our heritability findings raise the important question asking how the genetic variation underlying social relationships is associated with these biological mechanisms through which social factors mediate health.

### Twin relationship

Our results also echo those from other studies of the co-twin relationship showing that: (1) twin siblings generally have greater social closeness and are more dependent on each other than non-twin siblings, and (2) monozygotic twins tend to experience this closeness to a greater degree than do dizygotic twins (Fortuna et al. 2010; Johnson et al. 2002; Segal 1999; Segal et al. 2003). Compared to other siblings, co-twin relationships also tend to be more intense in several other respects, including frequency of contact, level of support or conflict, depth of intimacy (Neyer 2002).

The results of the variance decomposition analyses reveal important differences in the co-twin relationship compared to the other types of close relationships (Fig. 2) we investigated. Familial effects (heritability and common environment) were greatest for the twin relationship—for both support and for strain, and were more pronounced for males for strain but for females for support. In the case of the twin-relationship we emphasize the importance of familial effects rather than the compartmentalization of these into genetic from shared environmental influences. This is because these two sources of variation are highly confounded in the case of the twin relationship. Unique to the twin relationship is the situation where the relationship between the twins itself creates a shared environment to which both twins are equally exposed—albeit their response to that exposure will vary. Genetic factors can affect both the nature of the shared environment the twins create as well as their perception of support and strain within that relationship environment. Here, parallels can be drawn to other types of twin studies showing genetic influences on perceptions of family environment, where twins experiencing the same family at the same point in time will perceive those family environments quite differently (Plomin et al. 1988). An important implication of this is that individual perceptions may be important sources of non-shared environmental influences (Plomin and Daniels 1987, 2011). Treating the twin relationships separately, rather than having those ratings and perception embedded in a more general measure of family support and strain, is important in assess effects of the twin relationship uniquely and independently from other familial and sib relationships.

### Relationships between support and strain

Social support is negatively associated with social strain across all four sets of relationships we studied. Again, our findings mirror those reported with MIDUS and the relationship-specific values followed the same pattern of being lowest for friend support and friend strain, moderate for family support and family strain, and largest for spouse-support and spouse-strain (Walen and Lachman 2000). Estimates from bivariate models revealed that genetic effects explained between 8 and 40% of the phenotypic correlation. These are based on ACE/ADE models, the confidence intervals are large and results should be interpreted cautiously. However, results based on the more parsimonious models (Table 5 and S1) revealed little differences in the effects that mediate the relationship between support and strain with the exception of family support among women where genetic mediation of the relationship is attenuated in the fuller model. The findings for the twin and family relationship highlight the importance of shared environment, which explained just over 50% of the phenotypic correlation of twin support and twin strain or males and females and 25% of the phenotypic correlation of family support and family strain for males. Further, genetic effects accounted for little of the phenotypic relationship between support and strain in the twin and family relationship among the males. These findings cast new light on the nature of the co-twin and family relationship showing that the effects of the environment shared within these relationships overlap greatly between experiences of support and stress but exert their effects in the opposite direction.

To the best of our knowledge, our findings are the first to estimate the genetic and environmental sources of covariance between social support and social strain across close relationships. As evidenced by the genetic (rG) and non-shared environmental (rE) correlations there is large, but not complete, overlap between the genetic and environmental influences underlying variation in social support and social strain. Although we don’t know the specific nature of these influences, our findings suggest that a set of core factors exert inverse effects on experiences of support and strain. Health status and personality are two obvious realms where theory would predict that heritable variation and non-shared environmental influences could contribute to the inverse pattern of effects we observe. Another important point stemming from the bivariate results is that some of the genetic and non-shared environmental influences affecting variation in social support and strain are unique to either support or to strain. This has significant implications, particularly because social relationships have substantial effects on health, and having close ties is increasingly seen as a health behavior. If we are able to identify the factors that simultaneously and inversely influence social support and strain, and the factors that operate independently, then this could help the inform prevention and intervention strategies for behavior change and healthier aging (Lachman and Agrigoroaei 2010; Tesdahl and Gesell 2015).

## Limitations

A limitation of this study is that the support and strain measures are non-normally distributed. The best transformation according to the Box–Cox plot was dichotomizing of these variables (except spouse strain for which a square root transformation seemed to be the most plausible). This is discussed in detail elsewhere (Kutschke et al. 2016). Applying dichotomization to our measures would lead to a large loss of information from individuals who report modest levels of support and strain as these would have to be recoded either as low support/strain or high support/strain. Another analysis approach would be to treat the data as ordinal. However, each of the support/strain measures has between 18 and 20 unique values (levels) and such analysis is rather computationally heavy, and OpenMx did not manage to find a model which would satisfy global optimum criteria for these measures and standard errors were large and not trustworthy. Therefore, we opted to analyze the non-transformed continuous measures to facilitate comparisons with other studies; the skewed distributions could affect both the model fit statistics and lead to underestimates of C in an ACE model, and underestimates of A with overestimates of D and E in an ADE model (Derks et al. 2004).

Our analyses of the sources of covariation between support and strain revealed low power to drop familial effects. Larger samples are needed to derive more precise estimates of the genetic and environmental sources of covariation between these measures.

## Conclusion

Our study enhances existing research on social relationships in several important ways, including a systematic variance component analysis, reporting raw and standardized results, on four types of close relationships, and bivariate analyses of social support and social strain across close relationships. Importantly, the twin relationship was included as a separate type of relationship not combined within the general family category. Although the genetic and shared environmental influences are confounded in biometric models analyzing the twin relationship, the finding of greater familial effects for this relationship suggest that it is qualitatively different from other types of relationships regarding factors that influence variation in social support and strain. Our findings also reveal that genetic and shared environmental factors are important for explaining variation in the patterning of close relationships during adulthood, and there is large degree of overlap in the genetic effects that exert influence in opposite directions on measures of social support and social strain.

These findings are mainly descriptive, and just the first step in exploring the genetic and environmental underpinnings of social support and strain measures. On the basis of our results, a logical next analysis will be to explore the genetic and environmental sources of covariation within and across the support and strain domains. This will contribute importantly to our understanding the degree to which the same sets of genetic and environmental influences are important across different types of close relationships.

## Electronic supplementary material

Below is the link to the electronic supplementary material.

Supplementary material 1 (DOCX 17 KB)
