# Supplementary material for: Social Support and Strain Across Close Relationships: A Twin Study
Source: Behav Genet. 2018 Apr 12;48(3):173–86. doi: 10.1007/s10519-018-9899-x (PMC5934464; doi:10.1007/s10519-018-9899-x)
Supplement: Supplementary file 1 — Supplementary material 1 (DOCX 17 KB) [file 10519_2018_9899_MOESM1_ESM.docx]

# Supplementary material

Table S.1. Parameter estimates and (95% confidence intervals) from the best-fitting bivariate models accounting for age effects on means and heterogeneity across sex.

|  | **Support** | | | | | | **Strain** | | | | | | **rG** | **rD** | **rC** | **rE** | **rP** | **% of rP explained by:** | | | |
| --- | --- | --- | --- | --- | --- | --- | --- | --- | --- | --- | --- | --- | --- | --- | --- | --- | --- | --- | --- | --- | --- |
|  | **Mean** | **Age** | **St A** | **St D** | **St C** | **St E** | **Mean** | **Age** | **St A** | **St D** | **St C** | **St E** |  |  |  |  |  | **a** | **d** | **c** | **e** |
| Twin: support - ACE males, ACE females; strain - ACE males, ACE females | | | | | | | | | | | | | | | | | | | | | |
| m | 3.41  (3.37;  3.45) | -0.06  (-0.10;  -0.02) | 0.58  (0.41;  0.72) |  | 0.11  (0.00;  0.26) | 0.31  (0.27;  0.37) | 1.55  (1.52;  1.59) | -0.02  (-0.05;  0.02) | 0.24  (0.03;  0.45) |  | 0.27  (0.09;  0.44) | 0.49  (0.42;  0.56) | -0.05  (-0.37;  0.39) |  | -0.6 | -0.28  (-0.37;  -0.19) | -0.23  (-0.28;  -0.18) | 8 |  | 45 | 48 |
| f | 3.65  (3.62;  3.68) | -0.04  (-0.07;  -0.01) | 0.32  (0.21;  0.43) |  | 0.42  (0.31;  0.51) | 0.26  (0.23;  0.30) | 1.66  (1.63;  1.70) | -0.04  (-0.07;  -0.01) | 0.42  (0.29;  0.55) |  | 0.22  (0.10;  0.34) | 0.36  (0.32;  0.40) | -0.37  (-0.53;  -0.18) |  | -0.8 | -0.24  (-0.31;  -0.17) | -0.45  (-0.49;  -0.41) | 30 |  | 54 | 16 |
| Spouse: support – AE males, AE females; strain – AE males, AE females | | | | | | | | | | | | | | | | | | | | | |
| m | 3.76  (3.74;  3.79) | -0.00  (-0.03;  0.02) | 0.29  (0.19;  0.38) | 0 | 0 | 0.71  (0.62;  0.81) | 2.05  (2.02;  2.09) | -0.02  (-0.05;  0.02) | 0.36  (0.27;  0.45) | 0 | 0 | 0.64  (0.55;  0.73) | -0.59  (-0.75;  -0.41) | 0 | 0 | -0.42  (-0.50;  -0.34) | -0.48  (-0.52;  -0.43) | 40 | 0 | 0 | 60 |
| f | 3.65  (3.63;  3.68) | -0.01  (-0.03;  0.02) | 0.16  (0.05;  0.25) | 0 | 0 | 0.84  (0.75;  0.95) | 2.04  (2.01;  2.07) | -0.00  (-0.03;  0.03) | 0.25  (0.16;  0.34) | 0 | 0 | 0.75  (0.66;  0.84) | -0.77  (-1.00;  -0.53) | 0 | 0 | -0.46  (-0.52;  -0.38) | -0.52  (-0.55;  -0.48) | 29 | 0 | 0 | 71 |
| Family: support – ACE males, AE females; strain – CE males, AE females | | | | | | | | | | | | | | | | | | | | | |
| m | 3.49  (3.46;  3.52) | 0.01  (-0.02;  0.04) | 0.23  (0.00;  0.32) |  | 0.05  (0.00;  0.24) | 0.73  (0.64;  0.82) | 1.66  (1.63;  1.69) | -0.04  (-0.07;  -0.01) | 0 |  | 0.19  (0.12;  0.19) | 0.81  (0.81;  0.88) | 0 |  | -0.83  (-1.00;  -0.22) | -0.30  (-0.37;  -0.23) | -0.31  (-0.35;  -0.26) | 0 |  | 24 | 74 |
| f | 3.55  (3.53;  3.58) | 0.02  (-0.00;  0.04) | 0.36  (0.29;  0.42) |  | 0 | 0.64  (0.58;  0.71) | 1.76  (1.73;  1.78) | -0.07  (-0.09;  -0.04) | 0.38  (0.32;  0.44) |  | 0 | 0.62  (0.56;  0.68) | -0.54  (-0.65;  -0.42) |  | 0 | -0.30  (-0.37;  -0.24) | -0.39  (-0.43;  -0.35) | 51 |  | 0 | 49 |
| Friends: support – AE males, ADE females; strain – AE males, AE females | | | | | | | | | | | | | | | | | | | | | |
| m | 3.28  (3.25;  3.31) | -0.06  (-0.09;  -0.03) | 0.30  (0.22;  0.39) | 0 | 0 | 0.70  (0.61;  0.78) | 1.56  (1.53;  1.59) | -0.03  (-0.06;  -0.01) | 0.24  (0.15;  0.33) | 0 | 0 | 0.76  (0.67;  0.85) | -0.34  (-0.57;  -0.11) | 0 | 0 | -0.11  (-0.19;  -0.02) | -0.17  (-0.22;  -0.12) | 54 | 0 | 0 | 46 |
| f | 3.50  (3.47;  3.52) | -0.05  (-0.07;  -0.03) | 0.04  (0.01;  0.38) | 0.33  (0.00;  0.40) | 0 | 0.63  (0.63;  0.70) | 1.56  (1.53;  1.59) | -0.03  (-0.06;  -0.01) | 0.36  (0.29;  0.42) | 0 | 0 | 0.64  (0.58;  0.71) | -0.91  (-1.00;  -0.24) | 0 | 0 | -0.26  (-0.33;  -0.19) | -0.27  (-0.31;  -0.23) | 40 | 0 | 0 | 60 |

Note: m = males; f = females

St A = standardized additive genetic variance; St D = standardized dominant genetic variance; St C = standardized shared environmental variance; St E = standardized specific environmental variance

rG = additive genetic correlation; rD = dominant genetic correlation; rC = shared environmental correlation; rE = specific environmental correlation

rP = phenotypic correlation
